# Adaptive Immunity and the Risk of Autoreactivity in COVID-19

**DOI:** 10.3390/ijms22168965

**Published:** 2021-08-20

**Authors:** Rhiane Moody, Kirsty Wilson, Katie L. Flanagan, Anthony Jaworowski, Magdalena Plebanski

**Affiliations:** 1 School of Health and Biomedical Science, RMIT University, Bundoora, VIC 3083, Australia; s3740510@student.rmit.edu.au (R.M.); kirsty.wilson2@rmit.edu.au (K.W.); katie.flanagan@ths.tas.gov.au (K.L.F.); anthony.jaworowski@rmit.edu.au (A.J.); 2 Tasmanian Vaccine Trial Centre, Clifford Craig Foundation, Launceston General Hospital, Launceston, TAS 7250, Australia; 3 School of Medicine, University of Tasmania, Launceston, TAS 7250, Australia; 4 Department of Immunology and Pathology, Monash University, Melbourne, VIC 3004, Australia

**Keywords:** COVID-19, SARS-CoV-2, autoimmunity, autoantibodies, molecular mimicry

## Abstract

While first and foremost considered a respiratory infection, COVID-19 can result in complications affecting multiple organs. Immune responses in COVID-19 can both protect against the disease as well as drive it. Insights into these responses, and specifically the targets being recognised by the immune system, are of vital importance in understanding the side effects of COVID-19 and associated pathologies. The body’s adaptive immunity recognises and responds against specific targets (antigens) expressed by foreign pathogens, but not usually to target self-antigens. However, if the immune system becomes dysfunctional, adaptive immune cells can react to self-antigens, which can result in autoimmune disease. Viral infections are well reported to be associated with, or exacerbate, autoimmune diseases such as multiple sclerosis (MS) and systemic lupus erythematosus (SLE). In COVID-19 patients, both new onset MS and SLE, as well as the occurrence of other autoimmune-like pathologies, have been reported. Additionally, the presence of autoantibodies, both with and without known associations to autoimmune diseases, have been found. Herein we describe the mechanisms of virally induced autoimmunity and summarise some of the emerging reports on the autoimmune-like diseases and autoreactivity that is reported to be associated with SARS-CoV-2 infection.

## 1. Introduction

Since the emergence of the severe acute respiratory syndrome coronavirus 2 (SARS-CoV-2) in December 2019, as of 16 August 2021, there have been more than 207.1 million confirmed cases of COVID-19 and more than 4.3 million associated deaths [1]. Additionally, as of 16 August 2021, over four billion vaccine doses have been administered worldwide [1]. COVID-19 is commonly characterised with a sore throat, dry cough, fever and loss of taste or smell [2,3,4]. However, it is a multi-organ disease resulting in complications (such as acute injuries or abnormal tests) in the heart [5], gastrointestinal tract [6] and nervous system [7,8,9], as examples. These complications have additionally been autoimmune-like, suggesting the immune system’s potential role in pathology. 

The immune system is a highly regulated entity which functions to recognise and eliminate foreign material including infections and tumours. The effector cells involved in adaptive immunity are comprised of B and T lymphocytes, cells which express unique receptors on their surface which recognise specific regions of antigens called epitopes [10]. Epitopes between B and T cells differ. B cells will directly recognise free, exposed antigens. In contrast, T cells recognise a complex consisting of antigen peptide fragments within a molecule known as molecular histocompatibility complex (MHC), which is presented by other cells. Due to the diverse repertoire of receptors created during development, these epitopes may be self-antigens (autoantigens). The present review focuses on adaptive immunity, with an emphasis on B cells, as well as autoreactivity (attacking of self) that occurs during, or as a long-term consequence of, exposure to SARS-CoV-2 or its component macromolecules.

In COVID-19, B and T cell responses to the SARS-CoV-2 proteins have been studied in acute and convalescent infections [11,12]. Serological assays to measure the increased presence of antibodies to the spike and nucleoprotein have been used to 1. understand seroconversion (the appearance of specific antibodies), 2. identify neutralising antibodies (i.e., those that prevent viral infection of host cells) and 3. correlate the immune response with disease severity (systematically reviewed in [11]). There have also been reports of increased antibody responses to other SARS-CoV-2 proteins (such as open reading frame (ORF)3b and ORF8) after infection [13]. Similarly, T cell responses to multiple SARS-CoV-2 proteins have been identified [14], and responses elicited in COVID-19 patients have been studied (systemically reviewed in [12]). By studying and measuring T cell responses, insights into the role of T cells for the resolution of primary infection, as well as the establishment of long-term immunological memory able to react effectively to subsequent infections, can be gained. This is therefore also key for the development of both therapeutic and vaccine strategies. 

## 2. Viral Infections and Autoimmunity

A key pillar of the adaptive immune system is its ability to recognise and react to external pathogens (such as SARS-CoV-2), but not to self-antigens. This is controlled by immune tolerance, mechanisms which regulate an immune response, as well as the built-in unresponsiveness of a lymphocyte when its antigen-specific receptor engages with a cognate self-antigen [15,16]. Tolerance is particularly important in the context of self-antigens since it is undesirable to have an immune response to self. High frequencies of self-reactive immune cells can be found circulating in our bodies, although they usually remain suppressed [17,18,19]. These self-reactive immune cells can be activated by the dysregulation of immune tolerance mechanisms [20] or through inflammatory signals [21,22]. This can result in the immune system going awry, resulting in immunopathology and autoimmune diseases such as systemic lupus erythematosus (SLE) or multiple sclerosis (MS) [23,24].

**Figure 1:**
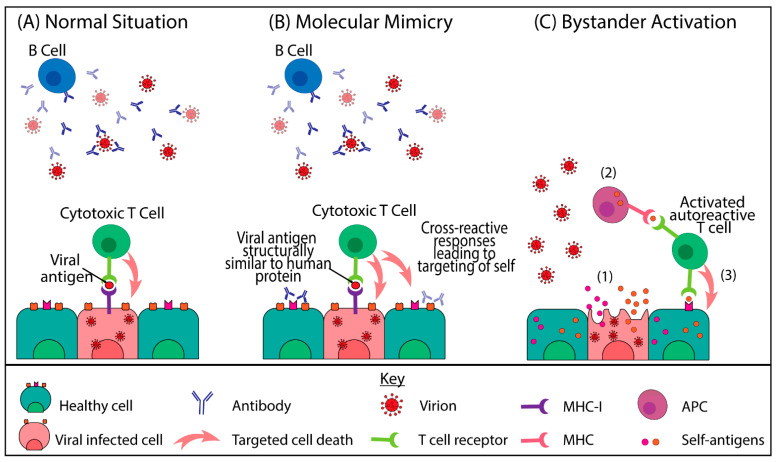
Mechanisms of virally induced autoimmunity. (**A**) In the normal situation, B cells will release antibodies upon activation that can bind to the extracellular virus [27]. Infected cells will present viral antigens on MHC class 1 (MHC-I) to cytotoxic T cells, resulting in the activation of the T cells and killing of these virus-infected cells [28]. (**B**) Molecular mimicry occurs when the viral antigen is structurally similar to human-derived proteins [26]. Antibodies may bind to both viruses and self-proteins (e.g., on healthy cell surfaces). T cells may become activated by the viral protein and target a virus-infected cell but also recognise and attack self. (**C**) Bystander activation. (1) Release of self-antigens into an inflammatory environment. (2) Antigen uptake and presentation by an antigen presenting cell (APC) to an autoreactive T cell. (3) Activated autoreactive T cell recognises and attacks healthy cells.

The triggering of autoimmune diseases is defined by a ’mosaic of autoimmunity’, a term that describes the combination of multiple contributory factors [25]. These factors can be grouped into the following four groups: genetic predisposition, immune defects, hormonal factors and environmental factors [25]. Amongst the environmental factors, viral infections are known to promote and exacerbate autoimmune diseases. Two of the key mechanisms proposed for viral-induced autoimmunity include molecular mimicry and bystander activation (Figure 1) [26]. Molecular mimicry occurs when the same lymphocyte receptor recognises both a foreign pathogen antigen and a self-protein due to their structure similarity, which can result in immune cross-reactivity. In contrast, bystander activation occurs when autoreactive immune cells become activated due to the liberation of self-antigens which are otherwise not exposed to the immune system. 

Associations between various viral infections and autoimmune diseases have been reported repeatedly in the literature (reviewed by Smatti et al. [26]). Active human cytomegalovirus (HCMV) infection is often found in patients diagnosed with immune thrombocytopenic purpura (ITP, an autoimmune blood disorder), and HCMV infection results in a more severe form of this autoimmune disease which is also resistant to treatment [29]. Antibody responses to HCMV have been found to be significantly elevated in SLE patients in comparison to healthy controls [30]. Another virus with a reported association with SLE is the Epstein−Barr virus (EBV) [31,32]. In comparison to healthy controls, EBV viral burden is abnormally elevated in SLE patients [31], and they have higher titres of anti-EBV antibodies [32]. High titres of anti-EBV antibodies are also present in rheumatoid arthritis (RA) patients [32]. Other examples of viral infections linked to autoimmune diseases include enteroviruses (e.g., coxsackievirus A4, coxsackievirus A2 and coxsackievirus A16) with islet autoimmunity [33] and measles, mumps and rubella with type 1 diabetes [34]. 

Several coronaviruses have additionally been linked to autoimmunity. Two of the common human coronaviruses, HCoV-229E and HCoV-OC43, have been linked with autoimmunity, specifically MS [35,36,37,38]. Antibodies to HCoV-OC43 and HCoV-229E were found intrathecally in 41% and 26% in people with MS, respectively [35]. This was in comparison to control subjects where no antibodies to either virus were detected. In a separate study, viral RNA for HCoV-229E was detected in central nervous system tissue in 36% of MS patients, but not in control subjects [36], suggesting a potential role of coronavirus infection in disease aetiology. Similarly, a statistically significant increase in the prevalence of viral RNA for HCoV-OC43 has been reported in MS patients in comparison to controls [37]. Furthermore, T cell immune cross-reactivity between myelin and HCoV-229E antigens has been reported in MS patients, in contrast to control subjects [38]. Of interest is a case report linking ITP to an infection with the common human coronavirus, HCoV-HKU1 [39], although more evidence will need to be accumulated to establish this association. Thrombocytopenia, which occurred in patients following infection with the previous epidemic causing coronavirus, SARS-CoV-1, has been suggested to be caused by an immune mechanism [40,41]. Another association between SARS-CoV-1 and autoimmune diseases has been identified through immune cross-reactivity [42]. Patients with autoimmune diseases (SLE, Sjögren’s syndrome, RA and mixed connective tissue disease) tested positive for antibodies to SARS-CoV-1 antigen, despite no previous SARS-CoV-1 infection [42]. Given these associations between coronaviruses and autoimmunity, as well as the sequence similarity of SARS-CoV-2 to these viruses, a link between SARS-CoV-2 and autoimmunity is plausible.

## 3. Autoantibodies Identified in COVID-19 Positive Patients

COVID-19 positive patients with more severe disease have increased levels of autoantibodies, including those that have known associations with autoimmune diseases [43,44,45,46,47,48]. One of the earliest studies showing this autoimmune phenomenon in severe COVID-19 cases investigated the antibody responses to 12 autoimmune-related targets in a cohort of 21 severe and critical patients [43]. Of these 12 antigen targets, 5 antigens were targeted in at least one patient; antinuclear antigen (ANA) antibodies (50%), anti-60 kDa SSA/Ro antibodies (25%), anti-52 kDa SSA/Ro antibodies (20%), anti-scl-70 antibodies (5%) and anti-U1-RNP antibodies (5%). Similarly, ANA antibodies were reported in 34.5% of severely ill COVID-19 cases in a separate cohort [44]. Within this second cohort, it was stated that no patients had a history of systemic autoimmunity, yet nearly 70% had autoantibodies relating to at least one systemic autoimmune rheumatic disease [44]. Amongst this cohort, anti-phospholipid antibodies (aPLs) were also common (cardiolipin (CL) and β2 glycoprotein I (β2GPI), 24.1% and 34.5%, respectively). APLs associated with antiphospholipid syndrome (an autoimmune disorder that can result in a variety of symptoms such as blood clots and chronic headaches) have been reported to be associated with SARS-CoV-2 infection in several studies [46,47,48,49,50]. Two studies identified that more than 50% of their subjects had antibodies to at least one type of phospholipid [46,48]. One of these reported that higher titres of aPLs were associated with more severe disease [46] whereas the other reported that thrombosis events only occurred in the aPL positive patients but not those without any detectable aPL. In contrast, Gatto et al. [50] found no association between aPL positivity and thrombosis among the patients they studied. Therefore, the role of these autoantibodies in co-morbid events in COVID-19 patients is still unclear. 

In addition to autoantibodies with known associations to autoimmune diseases, other autoantibodies to self-proteins, including cytokines and nervous system-related proteins, have also been found in COVID-19 patients [45,47]. Type I interferons (IFNs) are key cytokines in anti-viral immune responses [51]. The presence of autoantibodies to type I IFNs (-ω, -α or both) have been reported in 13.7% of patients with life threatening COVID-19 pneumonia [45]. These autoantibodies were specific to severe disease and not found in any COVID-19 patients with asymptomatic or mild infection. In 10.2% of patients with detectable anti-IFN antibodies, the antibodies had neutralising capabilities and were shown to neutralise the corresponding IFN’s ability to block SARS-CoV-2 infections in vitro [45]. Additionally, 15 (11.1%) of the 135 subjects positive for at least one type of anti-type I IFN also had autoantibodies to other cytokines including: IFN-γ, GM-CSF, IL-6, IL-10 and/or others [45]. Only in 4 of these 15 did the autoantibodies to other cytokines have neutralising capabilities, thus demonstrating that not all autoantibodies have potentially pathogenic roles. Furthermore, some patients presenting with neurological symptoms in severe COVID-19 have autoantibodies to neuronal targets [47]. In a cohort of 11 patients, anti-Yo antibodies were found in the serum and cerebral spinal fluid (CSF) of one patient, anti-myelin antibodies in the serum of two patients, and one patient had high levels of anti-NMDA receptor antibodies [47]. Three separate patients in this study were found to have aPLs [47].

The presence of autoantibodies highlights the state of dysregulation of the immune system in SARS-CoV-2 infection, particularly in severe cases. With COVID-19 presenting as a multi-organ disease, these autoantibodies are hypothesised to be playing a role in the pathology. However, in some cases, such as for anti-phospholipids and thrombosis, this remains unknown and further research into the role of these autoantibodies is required. 

## 4. Autoimmunity Associated with SARS-CoV-2

In addition to the presence of autoantibodies in COVID-19 positive patients, there have been multiple case reports of COVID-19-associated autoimmune diseases. Such examples include, but are not limited to, ITP, Guillain-Barré syndrome (GBS), SLE, MS, systemic rheumatoid diseases and multisystem inflammatory syndrome in children (MIS-C).

### 4.1. Immune Thrombocytopenia and Vasculitis Post Infection or Vaccination

Immune thrombocytopenia is a rare autoimmune disease characterised by low levels of platelets and therefore an increased risk of bleeding. Immune thrombocytopenia is a complication of COVID-19 that is found in both severe and non-severe disease, although at higher rates in the former [52]. There have been reports of patients with low platelet counts [53,54], with one patient reported to have autoantibodies to platelets themselves [53]. These patients additionally presented with skin lesions such as haematomas, petechiae [53] or purpura [54]. Moreover, it was reported that ITP occurred during active COVID-19 but also up to 10 days after COVID-19 symptoms ceased [53]. Furthermore, there have been reports of vasculitis, an autoimmune disease involving inflammation and narrowing of blood vessels. Presentations of leucocytoclastic vasculitis [55] and large vessel vasculitis [56] have been reported either as a manifestation of SARS-CoV-2 infection [55] or developed post-infection [56].

In addition to the SARS-CoV-2 infection associated immune thrombocytopenia, vaccine induced prothrombotic immune thrombocytopenia (VIPIT) has occurred following vaccination with ChAdOx1 nCoV-19 (AstraZeneca, Cambridge, United Kingdom) or Ad26.COV2.S (Johnson & Johnson, NJ, USA) COVID-19 vaccines [57,58,59]. Implicated in VIPIT is the presence of autoantibodies targeting platelet factor 4 (PF4) [57,58,59], which, along with thrombosis and/or thrombocytopenia, are part of the VIPIT diagnostic criteria [58]. It is suggested that VIPIT is similar to autoimmune-heparin induced thrombocytopenia (aHIT) [60], a disease where anti-PF4 autoantibodies are implicated in aetiology [61]. However, anti-PF4 autoantibodies have been reported in low numbers of individuals post-vaccination without associated VIPIT [62,63], suggesting that these autoantibodies are not the sole cause behind VIPIT aetiology. Further research is required into the mechanisms inducing anti-PF4 autoantibodies post-vaccination and their role in immune thrombocytopenia.

### 4.2. Autoimmune Haemolytic Anaemia and Cold Agglutinin Syndrome

Both autoimmune haemolytic anaemia (AIHA), a rare blood condition characterised by the presence of autoantibodies to red blood cells, and cold agglutinin syndrome, a form of AIHA characterised by anti-red blood cell agglutination at low temperatures, have been reported in COVID-19 patients [64,65,66,67]. Across these reports, consisting of a total of 11 cases, 54.5% were positive for cold agglutinin antibodies [64,65,66,67], 46.4% of patients with anti-erythrocyte antibodies diagnosed with warm AIHA [66] and 9.1% were positive for both cold agglutinin antibodies and anti-globulin antibodies [67]. The onset of AIHA (either warm or cold) is reported to range from 4–13 days following the onset of COVID-19 symptoms [64,66]. 

### 4.3. Guillain−Barré Syndrome and Miller Fisher Syndrome 

GBS, an autoimmune disease that attacks nerves, and Miller Fisher syndrome (MFS), a variant of GBS, were some of the earlier autoimmune-like complications reported in COVID-19 patients [68,69,70,71]. One correspondence reported the symptoms of GBS, including lower-limb weakness, paraesthesia (pins and needles) and ataxia (impaired coordination, balance and speech), occurring in five patients 5–10 days following onset of COVID-19 symptoms [68]. Similarly, a case study reported a patient who, 12 days post clearing a 5-day cough and fever, experienced numbness and paraesthesia in their extremities [69]. Over the following 10 days, this developed into distal-limb weakness and severe gait impairment. Although testing negative for SARS-CoV-2 infection by polymerase chain reaction (PCR), the patient was positive for anti-SARS-CoV-2 IgG antibodies, indicating post-COVID-19 disease. Two separate case reports have described the occurrence of MFS post COVID-19. The first described a patient with typical presentation of MSF (ophthalmoplegia (weakness of eyes muscles) and ataxia) 20 days after a positive SARS-CoV-2 test [70]. The second case report described presentation of paraesthesia and gait instability within a few days of developing a cough, fever and other symptoms [71].

### 4.4. Systemic Lupus Erythematosus, Multiple Sclerosis and Systemic Rheumatoid Disease

Case studies of new onset autoimmune diseases, SLE, MS and rheumatoid diseases have been reported in association with COVID-19. SLE, a multi-system autoimmune disease, has been reported both following [72] and accompanying [73] SARS-CoV-2 infection. Both cases report the presence of autoantibodies with known association to SLE, including anti-La, anti-CCP, anti-SSA/Ro [72], ANA and anti-dsDNA antibodies [73]. Some patients have also been found to meet the criteria for diagnosis of MS following COVID-19 [74,75,76]. In each of these cases, markers of autoimmunity (e.g., ANA, anti-La) as well as autoantibodies to neuronal targets aquaporin-4 and myelin oligodendrocyte glycoprotein were negative, suggesting that autoantibodies may not always be involved in the aetiologies of these COVID-19 related autoimmune-like conditions. Finally, a small incidence of systemic rheumatoid diseases post positive SARS-CoV-2 PCR results have been reported [77]. Among over 15,200 medical records examined, six cases of systemic rheumatoid disease were identified, each presenting differently [77]. Three of these occurred within a week of a positive SARS-CoV-2 test; one with inflammatory arthritis and one with giant cell arteritis. The third presented with severe proximal muscle weakness and was positive for anti-Mi2 and anti-TIF1γ autoantibodies. The other three cases all occurred more than two months after testing positive for SARS-CoV-2. The first case, diagnosed with antiphospholipid syndrome, subsequently tested positive in the lupus anticoagulant test, which persisted when retested a month later. The second case developed bilateral hand and wrist swelling, while the third case was diagnosed with primary Sjögren’s syndrome due to onset of sicca symptoms and the presence of ANA, anti-Ro52, anti-Ro60 and anti-ribonucleoprotein autoantibodies. 

### 4.5. Multisystem Inflammatory Syndrome in Children (MIS-C) 

Some children infected with SARS-CoV-2 present with severe multiorgan complications that overlap those found in Kawasaki disease, a self-limiting vasculitis thought to be triggered by viral infections and which has a well-reported presence of autoantibodies [78,79]. These overlapping symptoms include fever, conjunctivitis, lymphadenopathy (enlargement of lymph nodes), rashes, cardiovascular involvement and hypotension. In severe cases, cardiovascular shock and multi-organ failure can occur. In addition to these Kawasaki disease-like symptoms, symptoms such as shock and gastrointestinal symptoms, not typically seen in Kawasaki disease, are being reported with the SARS-CoV-2 associated disease [80]. Due to these differences, as well as the differences in age and ethnicity of impacted children (older children of non-Asian descent), this COVID-19 associated disease was termed the multisystem inflammatory syndrome in children (MIS-C) by the World Health Organisation [81]. Using large-scale screening techniques, autoantibodies to a range of targets have been reported in children diagnosed with MIS-C [82,83]. One study reported increased levels of autoantibodies to endoglin, a protein important for arterial structural integrity [82]. Other autoantibody targets that were specific to the MIS-C group include MAP2K2 and members of the casein kinase family [82]. Another study reported increased IgG autoantibodies to 189 targets and increased IgA to 108 targets [83]. Included amongst these were autoantibodies anti-La (associated with autoimmune diseases SLE and Sjögren’s disease) and anti-Jo-1 (antibodies associated with idiopathic inflammatory myopathies). However, this study reported that most targets of the autoantibodies in MIS-C have no connection to autoimmune diseases and instead report autoantibodies to targets associated with endothelial and cardiac tissues, the gastrointestinal tract and immune cell mediators, such as MUC15 and IFNγ-receptor 2 [83].

Many of these present as case reports, highlighting the presence of the autoimmune phenomena occurring post SARS-CoV-2 infection in only a small number of cases. Due to this, the causes and risk factors behind these autoimmune complications remain unknown. However, given the occurrence of these autoimmune complications, as well as other multi-organ pathologies, there has been some research into the potential mechanisms causing these complications. 

## 5. Viral Induced Autoimmune Mechanisms in COVID-19

Both bystander activation and, to a greater degree, molecular mimicry (Figure 1) have been proposed to be occurring in COVID-19 patients. While the manifestation of T cell bystander activation has been identified in COVID-19 patients [84,85], whether it results in autoreactivity has not been explored. In contrast, molecular mimicry has been hypothesised as the cause behind some pathologies (e.g., AIHA or vascular damage) observed in COVID-19 patients.

Thus far, there have only been a handful of studies assessing the sequence similarity between proteins in SARS-CoV-2 and the human proteome [86,87,88,89,90,91,92,93]. In these reports, a number of short (5–6 amino acid) sequences in SARS-CoV-2 proteins were found to be identical with a range of human-derived proteins [87,88,89,91,93]. Some of the identical sequences were then further located in SARS-CoV-2 immunogenic regions (regions likely to be recognised by the immune system). Most of these regions were recognised as B cell epitopes, though some potential T cell epitopes were also reported [88]. However, human-encoded proteins that share sequence similarities with SARS-CoV-2 proteins are limited in number. In these reports [87,88,89,91], the specific proteins described have been selected by the authors based on their research interests/protein families of interest, or as proteins localised in tissues whose targeting by antibodies or T cells could explain some of the pathologies observed in COVID-19 (Table 1).

Another bioinformatic approach to studying molecular mimicry is to screen predicted or known immune epitopes for identity or similarity to sequences in human proteins. Kanduc (2020) [86] used SARS-CoV-2 sequences identical to previously validated immunogenic regions from SARS-CoV-1 proteins to explore the potential for molecular mimicry. However, instead of focusing on a protein family, or one specific disease association, it was found that identical sequences could be found in human proteins that are associated with a range of disorders in different body tissues/systems (e.g., pulmonary, cardiac, neurological, vascular, etc.), thus demonstrating the large range of multi-organ pathologies that may arise due to immune cross-reactivity. In contrast, Lyons-Weiler (2020) [92] predicted potential epitopes in SARS-CoV-2 proteins using SVMTriP (a tool to predict B cell epitopes) and then compared these sequences to human proteins. Amongst 37 SARS-CoV-2 proteins, 8 were found to not have any immunogenic regions. Of the remaining proteins, all epitopes, except one nucleoprotein sequence, had sequences that correspond to human-derived proteins. Here, human proteins were represented within multiple cell types and tissues (e.g., B cells, brain, gastrointestinal tract, lungs, liver, etc.) that are associated with the adaptive immune system. 

These previously mentioned studies all used prediction-based approaches as ways to support the molecular mimicry hypothesis. One study, however, has shown that commercially available SARS-CoV-1 antibodies to the spike and nucleoprotein bind to both SARS-CoV-2 proteins and to human tissue antigens [94]. In this study, 50 different human antigens were tested and a range of strength of reactions between the commercial antibodies and human antigens was observed [94]. The antibody specific for the SARS-CoV-1 spike protein showed strong positive binding to the SARS-CoV-2 spike protein as well as to the human antigens transglutaminase 3 (tTG3), transglutaminase 2 (tTG2), α-myosin, collagen, claudin 5+6, and S100B. The nucleoprotein antibody reacted strongly with the SARS-CoV-2 nucleoprotein as well as the human proteins tTG6 and F-actin. Additionally, both spike and nucleoprotein antibodies had strong reactions with a series of human tissue proteins including extractable nuclear antigen (ENA), mitochondria M2, myelin basic protein (MBP), nuclear antigen (NA) and thyroid peroxidase (TPO). As this study was performed in the initial period of the SARS-CoV-2 outbreak, the authors have since reported results from a similar study but using monoclonal antibodies targeting the SARS-CoV-2 spike, nucleoprotein, membrane and envelope proteins [95]. These commercial antibody responses were measured to 55 human antigens from a range of tissues. Amongst these 55 proteins, the spike antibody reacted with 28, the nucleoprotein antibody reacted with 24, the membrane protein antibody reacted with 18 and the envelope targeting antibody reacted with 8. These reactivities ranged from weak to strong and cross-reactivity between targets was observed; for example, mitochondria M2 was found to be reactive with all four SARS-CoV-2 antibodies. 

Together, these studies demonstrate both in silico and in vitro sequence similarity between SARS-CoV-2 immunogenic regions and human proteins found in multiple organs/body systems. These findings support the molecular mimicry hypothesis, with immune cross-reactivity and the potential of a dysregulated immune system resulting in the multi-organ complications seen in COVID-19. 

## 6. Limitations and Future Directions

The occurrence of autoimmune phenomena during or post COVID-19 suggests SARS-CoV-2 infection results in the dysregulation of the immune system resulting in autoimmunity. Here, various types of autoimmune diseases linked to SARS-CoV-2 infection have been addressed. However, in several cases the clinical observations of autoimmunity have only been reported in small patient numbers or as single case studies. In the future, combining more studies and reports in a systematic review will provide a greater understanding of autoimmune trends occurring in association with SARS-CoV-2 infection. In addition, the presence of cross-reactivity between SARS-CoV-2 specific antibodies and self-antigens suggests molecular mimicry and bystander activation may indeed be playing a role in pathology. However, further laboratory-based studies are required, whether to validate the prediction-based studies of molecular mimicry, identify any T cell association with autoreactivity, or to further understand the mechanisms causing the COVID-19 associated autoimmunity and pathologies.

## 7. Conclusions

The COVID-19 pandemic is a continually changing situation. As a new infection with potentially novel pathologies, it is important to understand the biological and clinical phenomena being observed during and post infection or vaccination. Currently, clinical observations and laboratory studies indicate that SARS-CoV-2 causes dysregulation of immune responses which may associate SARS-CoV-2 infection and autoimmunity. Identifying mechanisms and the self-targets being recognised by the immune system, as well as following clinical case-reports, may give further insights into therapeutic and vaccine responses. Moreover, they will provide early insights on the associated autoimmune diseases that may arise in susceptible individuals.

## Figures and Tables

**Table 1:** Human proteins found with identical sequences to SARS-CoV-2 proteins based on protein families and disease associations.

Protein Family/Disease Association	Protein	Reference
Autoimmune haemolytic anaemia	Ankyrin 1 (ANK1)	[87]
Human molecular chaperones	17 proteins listed, including: heat shock proteins, DNAJ homologs, etc.	[88]
Pulmonary surfactant related proteins	23 proteins listed, e.g., Alpha-2A adrenergic receptor (ADA2A) Transcriptional termination factor 1 (TTF1)	[89]
Anosmia	Odorant Receptor 7D4 (OR7D4)	[90]
Leukopenia	Poly (ADP-Ribose) Polymerase Family Member 9 (PARP9)	[90]
Vascular damage	Solute Carrier Family 12 Member 6 (SLC12A6)	[90]
Brainstem (pre-Bözinger complex) proteins	Disabled Homolog 1 (DAB1) Apoptosis-inducing factor 1, mitochondrial (AIFM1) Surfeit locus protein 1	[91]

## Data Availability

Not applicable.

## References

[1] WHO Who Coronavirus Disease (COVID-19) Dashboard. https://covid19.who.int/.

[2] Huang C., Wang Y., Li X., Ren L., Zhao J., Hu Y., Zhang L., Fan G., Xu J., Gu X. (2020). Clinical features of patients infected with 2019 novel coronavirus in Wuhan, China. Lancet.

[3] WHO Coronavirus Disease (COVID-19) Advice for the Public. https://www.who.int/emergencies/diseases/novel-coronavirus-2019/advice-for-public.

[4] Meng X., Deng Y., Dai Z., Meng Z. (2020). COVID-19 and anosmia: A review based on up-to-date knowledge. Am. J. Otolaryngol..

[5] Han H., Xie L., Liu R., Yang J., Liu F., Wu K., Chen L., Hou W., Feng Y., Zhu C. (2020). Analysis of heart injury laboratory parameters in 273 COVID-19 patients in one hospital in wuhan, China. J. Med. Virol..

[6] Cheung S., Quiwa J.C., Pillai A., Onwu C., Tharayil Z.J., Gupta R. (2020). Superior mesenteric artery thrombosis and acute intestinal ischemia as a consequence of COVID-19 infection. Am. J. Case Rep..

[7] Nalleballe K., Reddy Onteddu S., Sharma R., Dandu V., Brown A., Jasti M., Yadala S., Veerapaneni K., Siddamreddy S., Avula A. (2020). Spectrum of neuropsychiatric manifestations in COVID-19. Brain Behav. Immun..

[8] Poyiadji N., Shahin G., Noujaim D., Stone M., Patel S., Griffith B. (2020). COVID-19–associated acute hemorrhagic necrotizing encephalopathy: Imaging features. Radiology.

[9] Oxley T.J., Mocco J., Majidi S., Kellner C.P., Shoirah H., Singh I.P., De Leacy R.A., Shigematsu T., Ladner T.R., Yaeger K.A. (2020). Large-vessel stroke as a presenting feature of COVID-19 in the young. N. Engl. J. Med..

[10] Sanchez-Trincado J.L., Gomez-Perosanz M., Reche P.A. (2017). Fundamentals and methods for t- and b-cell epitope prediction. J. Immunol. Res..

[11] Post N., Eddy D., Huntley C., van Schalkwyk M.C.I., Shrotri M., Leeman D., Rigby S., Williams S.V., Bermingham W.H., Kellam P. (2021). Antibody response to sars-cov-2 infection in humans: A systematic review. PLoS ONE.

[12] Shrotri M., van Schalkwyk M.C.I., Post N., Eddy D., Huntley C., Leeman D., Rigby S., Williams S.V., Bermingham W.H., Kellam P. (2021). T cell response to sars-cov-2 infection in humans: A systematic review. PLoS ONE.

[13] Hachim A., Kavian N., Cohen C.A., Chin A.W.H., Chu D.K.W., Mok C.K.P., Tsang O.T.Y., Yeung Y.C., Perera R.A.P.M., Poon L.L.M. (2020). Orf8 and orf3b antibodies are accurate serological markers of early and late sars-cov-2 infection. Nat. Immunol..

[14] Grifoni A., Weiskopf D., Ramirez S.I., Mateus J., Dan J.M., Moderbacher C.R., Rawlings S.A., Sutherland A., Premkumar L., Jadi R.S. (2020). Targets of t cell responses to sars-cov-2 coronavirus in humans with COVID-19 disease and unexposed individuals. Cell.

[15] Xing Y., Hogquist K.A. (2012). T-cell tolerance: Central and peripheral. Cold Spring Harb. Perspect. Biol..

[16] Nemazee D. (2017). Mechanisms of central tolerance for b cells. Nat. Rev. Immunol..

[17] Maeda Y., Nishikawa H., Sugiyama D., Ha D., Hamaguchi M., Saito T., Nishioka M., Wing J.B., Adeegbe D., Katayama I. (2014). Detection of self-reactive cd8(+) t cells with an anergic phenotype in healthy individuals. Science.

[18] Richards D.M., Ruggiero E., Hofer A.-C., Sefrin J.P., Schmidt M., von Kalle C., Feuerer M. (2015). The contained self-reactive peripheral t cell repertoire: Size, diversity, and cellular composition. J. Immunol..

[19] Meffre E., Wardemann H. (2008). B-cell tolerance checkpoints in health and autoimmunity. Curr. Opin. Immunol..

[20] Makkouk A., Weiner G.J. (2015). Cancer immunotherapy and breaking immune tolerance: New approaches to an old challenge. Cancer Res..

[21] Ohashi P.S., DeFranco A.L. (2002). Making and breaking tolerance. Curr. Opin. Immunol..

[22] Jackson S.R., Yuan J., Berrien-Elliott M.M., Chen C.L., Meyer J.M., Donlin M.J., Teague R.M. (2014). Inflammation programs self-reactive cd8+ t cells to acquire t-box-mediated effector function but does not prevent deletional tolerance. J. Leukoc. Biol..

[23] Zharkova O., Celhar T., Cravens P.D., Satterthwaite A.B., Fairhurst A.M., Davis L.S. (2017). Pathways leading to an immunological disease: Systemic lupus erythematosus. Rheumatology.

[24] Høglund R.A., Maghazachi A.A. (2014). Multiple sclerosis and the role of immune cells. World J. Exp. Med..

[25] De Carvalho J.F., Pereira R.M., Shoenfeld Y. (2009). The mosaic of autoimmunity: The role of environmental factors. Front. Biosci. (Elite Ed.).

[26] Smatti M.K., Cyprian F.S., Nasrallah G.K., Al Thani A.A., Almishal R.O., Yassine H.M. (2019). Viruses and autoimmunity: A review on the potential interaction and molecular mechanisms. Viruses.

[27] Dörner T., Radbruch A. (2007). Antibodies and b cell memory in viral immunity. Immunity.

[28] Halle S., Halle O., Förster R. (2017). Mechanisms and dynamics of t cell-mediated cytotoxicity in vivo. Trends Immunol..

[29] DiMaggio D., Anderson A., Bussel J.B. (2009). Cytomegalovirus can make immune thrombocytopenic purpura refractory. Br. J. Haematol..

[30] Chen J., Zhang H., Chen P., Lin Q., Zhu X., Zhang L., Xue X. (2015). Correlation between systemic lupus erythematosus and cytomegalovirus infection detected by different methods. Clin. Rheumatol..

[31] Moon U.Y., Park S.J., Oh S.T., Kim W.U., Park S.H., Lee S.H., Cho C.S., Kim H.Y., Lee W.K., Lee S.K. (2004). Patients with systemic lupus erythematosus have abnormally elevated epstein-barr virus load in blood. Arthritis Res..

[32] Yokochi T., Yanagawa A., Kimura Y., Mizushima Y. (1989). High titer of antibody to the epstein-barr virus membrane antigen in sera from patients with rheumatoid arthritis and systemic lupus erythematosus. J. Rheumatol..

[33] Honkanen H., Oikarinen S., Nurminen N., Laitinen O.H., Huhtala H., Lehtonen J., Ruokoranta T., Hankaniemi M.M., Lecouturier V., Almond J.W. (2017). Detection of enteroviruses in stools precedes islet autoimmunity by several months: Possible evidence for slowly operating mechanisms in virus-induced autoimmunity. Diabetologia.

[34] Ramondetti F., Sacco S., Comelli M., Bruno G., Falorni A., Iannilli A., d’Annunzio G., Iafusco D., Songini M., Toni S. (2012). Type 1 diabetes and measles, mumps and rubella childhood infections within the italian insulin-dependent diabetes registry. Diabet. Med..

[35] Salmi A., Ziola B., Hovi T., Reunanen M. (1982). Antibodies to coronaviruses oc43 and 229e in multiple sclerosis patients. Neurology.

[36] Stewart J.N., Mounir S., Talbot P.J. (1992). Human coronavirus gene expression in the brains of multiple sclerosis patients. Virology.

[37] Arbour N., Day R., Newcombe J., Talbot P.J. (2000). Neuroinvasion by human respiratory coronaviruses. J. Virol..

[38] Talbot P.J., Paquette J.S., Ciurli C., Antel J.P., Ouellet F. (1996). Myelin basic protein and human coronavirus 229e cross-reactive t cells in multiple sclerosis. Ann. Neurol..

[39] Magdi M., Rahil A. (2019). Severe immune thrombocytopenia complicated by intracerebral haemorrhage associated with coronavirus infection: A case report and literature review. Eur. J. Case Rep. Intern. Med..

[40] Wong R.S.M., Wu A., To K.F., Lee N., Lam C.W.K., Wong C.K., Chan P.K.S., Ng M.H.L., Yu L.M., Hui D.S. (2003). Haematological manifestations in patients with severe acute respiratory syndrome: Retrospective analysis. BMJ.

[41] Yang M., Ng M.H., Li C.K. (2005). Thrombocytopenia in patients with severe acute respiratory syndrome (review). Hematology.

[42] Wang Y., Sun S., Shen H., Jiang L., Zhang M., Xiao D., Liu Y., Ma X., Zhang Y., Guo N. (2004). Cross-reaction of sars-cov antigen with autoantibodies in autoimmune diseases. Cell Mol. Immunol.

[43] Zhou Y., Han T., Chen J., Hou C., Hua L., He S., Guo Y., Zhang S., Wang Y., Yuan J. (2020). Clinical and autoimmune characteristics of severe and critical cases of COVID-19. Clin. Transl. Sci..

[44] Vlachoyiannopoulos P.G., Magira E., Alexopoulos H., Jahaj E., Theophilopoulou K., Kotanidou A., Tzioufas A.G. (2020). Autoantibodies related to systemic autoimmune rheumatic diseases in severely ill patients with COVID-19. Ann. Rheum. Dis..

[45] Bastard P., Rosen L.B., Zhang Q., Michailidis E., Hoffmann H.-H., Zhang Y., Dorgham K., Philippot Q., Rosain J., Béziat V. (2020). Autoantibodies against type i ifns in patients with life-threatening COVID-19. Science.

[46] Zuo Y., Estes S.K., Ali R.A., Gandhi A.A., Yalavarthi S., Shi H., Sule G., Gockman K., Madison J.A., Zuo M. (2020). Prothrombotic autoantibodies in serum from patients hospitalized with COVID-19. Sci. Transl. Med..

[47] Franke C., Ferse C., Kreye J., Reincke S.M., Sanchez-Sendin E., Rocco A., Steinbrenner M., Angermair S., Treskatsch S., Zickler D. (2021). High frequency of cerebrospinal fluid autoantibodies in COVID-19 patients with neurological symptoms. Brain Behav. Immun..

[48] Zhang Y., Cao W., Jiang W., Xiao M., Li Y., Tang N., Liu Z., Yan X., Zhao Y., Li T. (2020). Profile of natural anticoagulant, coagulant factor and anti-phospholipid antibody in critically ill COVID-19 patients. J. Thromb. Thrombolysis.

[49] Zhang Y., Xiao M., Zhang S., Xia P., Cao W., Jiang W., Chen H., Ding X., Zhao H., Zhang H. (2020). Coagulopathy and antiphospholipid antibodies in patients with COVID-19. N. Engl. J. Med..

[50] Gatto M., Perricone C., Tonello M., Bistoni O., Cattelan A.M., Bursi R., Cafaro G., De Robertis E., Mencacci A., Bozza S. (2020). Frequency and clinical correlates of antiphospholipid antibodies arising in patients with sars-cov-2 infection: Findings from a multicentre study on 122 cases. Clin. Exp. Rheumatol..

[51] McNab F., Mayer-Barber K., Sher A., Wack A., O’Garra A. (2015). Type i interferons in infectious disease. Nat. Rev. Immunol..

[52] Bhattacharjee S., Banerjee M. (2020). Immune thrombocytopenia secondary to COVID-19: A systematic review. SN Compr. Clin. Med..

[53] Bomhof G., Mutsaers P., Leebeek F.W.G., Te Boekhorst P.A.W., Hofland J., Croles F.N., Jansen A.J.G. (2020). COVID-19-associated immune thrombocytopenia. Br. J. Haematol..

[54] Zulfiqar A.-A., Lorenzo-Villalba N., Hassler P., Andrès E. (2020). Immune thrombocytopenic purpura in a patient with COVID-19. N. Engl. J. Med..

[55] Camprodon Gómez M., González-Cruz C., Ferrer B., Barberá M.J. (2020). Leucocytoclastic vasculitis in a patient with COVID-19 with positive sars-cov-2 pcr in skin biopsy. BMJ Case Rep. CP.

[56] Oda R., Inagaki T., Ishikane M., Hotta M., Shimomura A., Sato M., Nakamoto T., Akiyama Y., Yamamoto K., Minamimoto R. (2020). Case of adult large vessel vasculitis after sars-cov-2 infection. Ann. Rheum. Dis..

[57] Scully M., Singh D., Lown R., Poles A., Solomon T., Levi M., Goldblatt D., Kotoucek P., Thomas W., Lester W. (2021). Pathologic antibodies to platelet factor 4 after chadox1 ncov-19 vaccination. N. Engl. J. Med..

[58] Greinacher A., Thiele T., Warkentin T.E., Weisser K., Kyrle P.A., Eichinger S. (2021). Thrombotic thrombocytopenia after chadox1 ncov-19 vaccination. N. Engl. J. Med..

[59] Muir K.-L., Kallam A., Koepsell S.A., Gundabolu K. (2021). Thrombotic Thrombocytopenia after Ad26.COV2.S Vaccination. N. Engl. J. Med..

[60] Schultz N.H., Sørvoll I.H., Michelsen A.E., Munthe L.A., Lund-Johansen F., Ahlen M.T., Wiedmann M., Aamodt A.-H., Skattør T.H., Tjønnfjord G.E. (2021). Thrombosis and Thrombocytopenia after ChAdOx1 nCoV-19 Vaccination. N. Engl. J. Med..

[61] Greinacher A., Selleng K., Warkentin T.E. (2017). Autoimmune heparin-induced thrombocytopenia. J. Thromb. Haemost..

[62] Sørvoll I.H., Horvei K.D., Ernstsen S.L., Laegreid I.J., Lund S., Grønli R.H., Olsen M.K., Jacobsen H.K., Eriksson A., Halstensen A.M. (2021). An observational study to identify the prevalence of thrombocytopenia and anti-pf4/polyanion antibodies in norwegian health care workers after COVID-19 vaccination. J. Thromb. Haemost..

[63] Thiele T., Ulm L., Holtfreter S., Schönborn L., Kuhn S.O., Scheer C., Warkentin T.E., Bröker B., Becker K., Aurich K. (2021). Frequency of positive anti-PF4/polyanion antibody tests after COVID-19 vaccination with ChAdOx1 nCoV-19 and BNT162b2. Blood.

[64] Jensen C.E., Wilson S., Thombare A., Weiss S., Ma A. (2020). Cold agglutinin syndrome as a complication of COVID-19 in two cases. Clin. Infect. Pract..

[65] Maslov D.V., Simenson V., Jain S., Badari A. (2020). COVID-19 and cold agglutinin hemolytic anemia. TH Open.

[66] Lazarian G., Quinquenel A., Bellal M., Siavellis J., Jacquy C., Re D., Merabet F., Mekinian A., Braun T., Damaj G. (2020). Autoimmune haemolytic anaemia associated with COVID-19 infection. Br. J. Haematol..

[67] Patil N.R., Herc E.S., Girgis M. (2020). Cold agglutinin disease and autoimmune hemolytic anemia with pulmonary embolism as a presentation of COVID-19 infection. Hematol. Oncol. Stem Cell Ther..

[68] Toscano G., Palmerini F., Ravaglia S., Ruiz L., Invernizzi P., Cuzzoni M.G., Franciotta D., Baldanti F., Daturi R., Postorino P. (2020). Guillain–barré syndrome associated with sars-cov-2. N. Engl. J. Med..

[69] Zito A., Alfonsi E., Franciotta D., Todisco M., Gastaldi M., Cotta Ramusino M., Ceroni M., Costa A. (2020). COVID-19 and guillain-barré syndrome: A case report and review of literature. Front. Neurol..

[70] Senel M., Abu-Rumeileh S., Michel D., Garibashvili T., Althaus K., Kassubek J., Otto M. (2020). Miller-fisher syndrome after COVID-19: Neurochemical markers as an early sign of nervous system involvement. Eur. J. Neurol..

[71] Gutiérrez-Ortiz C., Méndez-Guerrero A., Rodrigo-Rey S., San Pedro-Murillo E., Bermejo-Guerrero L., Gordo-Mañas R., de Aragón-Gómez F., Benito-León J. (2020). Miller fisher syndrome and polyneuritis cranialis in COVID-19. Neurology.

[72] Zamani B., Moeini Taba S.-M., Shayestehpour M. (2021). Systemic lupus erythematosus manifestation following COVID-19: A case report. J. Med. Case Rep..

[73] Mantovani Cardoso E., Hundal J., Feterman D., Magaldi J. (2020). Concomitant new diagnosis of systemic lupus erythematosus and COVID-19 with possible antiphospholipid syndrome. Just a coincidence? A case report and review of intertwining pathophysiology. Clin. Rheumatol..

[74] Palao M., Fernández-Díaz E., Gracia-Gil J., Romero-Sánchez C.M., Díaz-Maroto I., Segura T. (2020). Multiple sclerosis following sars-cov-2 infection. Mult. Scler. Relat. Disord..

[75] Moore L., Ghannam M., Manousakis G. (2021). A first presentation of multiple sclerosis with concurrent COVID-19 infection. Eneurologicalsci.

[76] Yavari F., Raji S., Moradi F., Saeidi M. (2020). Demyelinating changes alike to multiple sclerosis: A case report of rare manifestations of COVID-19. Case Rep. Neurol. Med..

[77] Hsu T.Y.T., D’Silva K.M., Patel N.J., Fu X., Wallace Z.S., Sparks J.A. (2021). Incident systemic rheumatic disease following COVID-19. Lancet Rheumatol..

[78] Jones V.G., Mills M., Suarez D., Hogan C.A., Yeh D., Segal J.B., Nguyen E.L., Barsh G.R., Maskatia S., Mathew R. (2020). COVID-19 and kawasaki disease: Novel virus and novel case. Hosp. Pediatr..

[79] Hicar M.D. (2020). Antibodies and immunity during kawasaki disease. Front. Cardiovasc. Med..

[80] Toubiana J., Poirault C., Corsia A., Bajolle F., Fourgeaud J., Angoulvant F., Debray A., Basmaci R., Salvador E., Biscardi S. (2020). Kawasaki-like multisystem inflammatory syndrome in children during the COVID-19 pandemic in paris, france: Prospective observational study. BMJ.

[81] Organisation W.H. Multisystem Inflammatory Syndrome in Children and Adolescents Temporally Related to COVID-19. https://www.who.int/news-room/commentaries/detail/multisystem-inflammatory-syndrome-in-children-and-adolescents-with-covid-19.

[82] Consiglio C.R., Cotugno N., Sardh F., Pou C., Amodio D., Rodriguez L., Tan Z., Zicari S., Ruggiero A., Pascucci G.R. (2020). The immunology of multisystem inflammatory syndrome in children with COVID-19. Cell.

[83] Gruber C.N., Patel R.S., Trachtman R., Lepow L., Amanat F., Krammer F., Wilson K.M., Onel K., Geanon D., Tuballes K. (2020). Mapping systemic inflammation and antibody responses in multisystem inflammatory syndrome in children (mis-c). Cell.

[84] Gregorova M., Morse D., Brignoli T., Steventon J., Hamilton F., Albur M., Arnold D., Thomas M., Halliday A., Baum H. (2020). Post-acute COVID-19 associated with evidence of bystander t-cell activation and a recurring antibiotic-resistant bacterial pneumonia. Elife.

[85] Bergamaschi L., Mescia F., Turner L., Hanson A.L., Kotagiri P., Dunmore B.J., Ruffieux H., De Sa A., Huhn O., Morgan M.D. (2021). Longitudinal analysis reveals that delayed bystander cd8+ t cell activation and early immune pathology distinguish severe COVID-19 from mild disease. Immunity.

[86] Kanduc D. (2020). From anti-sars-cov-2 immune responses to COVID-19 via molecular mimicry. Antibodies.

[87] Angileri F., Légaré S., Marino Gammazza A., Conway de Macario E., Macario A.J.L., Cappello F. (2020). Is molecular mimicry the culprit in the autoimmune haemolytic anaemia affecting patients with COVID-19?. Br. J. Haematol..

[88] Marino Gammazza A., Légaré S., Lo Bosco G., Fucarino A., Angileri F., Conway de Macario E., Macario A.J., Cappello F. (2020). Human molecular chaperones share with sars-cov-2 antigenic epitopes potentially capable of eliciting autoimmunity against endothelial cells: Possible role of molecular mimicry in COVID-19. Cell Stress Chaperones.

[89] Kanduc D., Shoenfeld Y. (2020). On the molecular determinants of the sars-cov-2 attack. Clin. Immunol..

[90] Angileri F., Legare S., Marino Gammazza A., Conway de Macario E., Jl Macario A., Cappello F. (2020). Molecular mimicry may explain multi-organ damage in COVID-19. Autoimmun. Rev..

[91] Lucchese G., Flöel A. (2020). Molecular mimicry between sars-cov-2 and respiratory pacemaker neurons. Autoimmun. Rev..

[92] Lyons-Weiler J. (2020). Pathogenic priming likely contributes to serious and critical illness and mortality in COVID-19 via autoimmunity. J. Transl. Autoimmun..

[93] Ehrenfeld M., Tincani A., Andreoli L., Cattalini M., Greenbaum A., Kanduc D., Alijotas-Reig J., Zinserling V., Semenova N., Amital H. (2020). COVID-19 and autoimmunity. Autoimmun. Rev..

[94] Vojdani A., Kharrazian D. (2020). Potential antigenic cross-reactivity between sars-cov-2 and human tissue with a possible link to an increase in autoimmune diseases. Clin. Immunol..

[95] Vojdani A., Vojdani E., Kharrazian D. (2021). Reaction of human monoclonal antibodies to sars-cov-2 proteins with tissue antigens: Implications for autoimmune diseases. Front. Immunol..

